# Assessment of Monkeypox (MPOX) Knowledge and Vaccination Intention among Health and Life Sciences Students in Algeria: A Cross-Sectional Study

**DOI:** 10.3390/idr16020013

**Published:** 2024-02-22

**Authors:** Mohamed Lounis, Ahmed Hamimes, Ali Dahmani

**Affiliations:** 1 Department of Agro-Veterinary Sciences, Faculty of Natural and Life Sciences, University of Ziane Achour, BP 3117, Road of Moudjbara, Djelfa 17000, Algeria; 2 Laboratoire d’Exploration e et Valorisation des Écosystèmes Steppiques, Faculty of Natural and Life Sciences, University of Ziane Achour, BP 3117, Road of Moudjbara, Djelfa 17000, Algeria; 3 Laboratory of Biostatistics, Bioinformatics and Mathematical Methodology Applied to Health Sciences (BIOSTIM), Faculty of Medicine, University of Constantine 3, Constantine 25000, Algeria; ahmed.hamimes@univ-constantine3.dz; 4 Institute of Veterinary Sciences, University of Blida 1, Blida 09000, Algeria; dahmanivet4@gmail.com

**Keywords:** monkeypox, knowledge, vaccine hesitancy, health and life science students, Algeria

## Abstract

Monkeypox (MPOX) is a viral zoonotic disease affecting endemically the Central and Western regions of Africa. The ongoing outbreak in non-endemic countries has made this disease a global concern. While no cases have been reported in Algeria, it is important to raise awareness about the disease to prepare for a potential outbreak, especially in light of the cases reported in neighboring Middle East and North African (MENA) countries. This study aimed to evaluate the knowledge and attitude of Algerian Health and Life Sciences students toward MPOX and its vaccine through an anonymous online survey. A total of 196 students participated in this study. Students of medicine (64.3%), females (85.7%), and those under 20 years of age (55.1%) were the most represented. The results revealed a low level of knowledge represented by a score of only 42.8% for correct answers with multiple gaps in epidemiology, etiology, and clinical manifestations of MPOX. Students of veterinary sciences showed the highest levels of knowledge (OR: 6.71; CI95%: 1.23–36.77), while those aged between 20 and 30 years old (OR: 0.11; CI95%: 0.02–0.79) and those vaccinated against seasonal flu (OR: 0.42; CI95%: 0.21–0.85) were associated with low levels of knowledge. Regarding MPOX vaccination, the study found a moderate level of acceptance (48.5%) among the surveyed students with Natural and Life Sciences students and those having a high vaccine conspiracy belief score (VCBS) showing the lowest level of acceptance. These findings highlight the need for educational programs and intensified public awareness campaigns to improve knowledge about MPOX and emphasize the importance of vaccination in preventing outbreaks and overcoming vaccine reluctance.

## 1. Introduction

Occurring endemically in the Central and Western region of Africa since the end of the seventies (according to World Health Organization (WHO)), human monkeypox (MPOX) has also emerged in non-endemic countries such as the USA (2003 and 2021), the UK (2018–2019 and 2021), and Singapore (2019). These outbreaks were generally geographically limited and mostly linked with travel to Africa [1,2]. However, in May 2022, MPOX has surprisingly re-emerged in multiple non-endemic countries. The particular facts of these outbreaks are the instantaneous reports of multiple cases in many countries, mainly in Europe and the USA, in addition to the absence of a link with traveling to endemic countries [3]. This situation prompted the WHO to declare the illness a Public Health Emergency of International Concern (PHEIC) on 23 July 2022 [4].

As of 17 March 2023, approximately 86,601 individuals were affected with 112 deaths in non-endemic regions [5]. It is noteworthy that these numbers reflected a case fatality rate (CFR) lower than those reported in African endemic countries, varying from 3.6 to 10.6% [1], and could be as low as 0.03% [6]. Furthermore, the case hospitalization rate (CHR) was estimated at 14.1% with a variation from 5.8% (during the year 2022) to 49.8% (before the year 2017) [6].

Transmission occurs generally after close contact with affected humans or animals. Cutaneous or mucosal lesions, blood, body fluids, respiratory droplets, and contaminated materials such as bedding are the main sources of contamination [7]. The clinical features of the disease include an incubation period of 6 to 13 days (range: 5 to 21 days), and the development of self-limiting lesions, skin nodules, or a disseminated rash, that generally last for 2 to 4 weeks. In addition, fever, headaches, back discomfort, low energy, muscle soreness, and enlarged lymph nodes are the most common signs reported in the current outbreak of MPOX [7,8]. These signs recall smallpox symptoms with less severity and better outcomes [9]. Consequently, in the absence of specific vaccines against this disease, some new-generation smallpox vaccines (MVA-BN-JYNNEOS, LC16, and ACAM2000) have been advised for high-risk individuals in some countries [2,8].

Although not to the same extent, the health community is nevertheless faced with a new challenge to convince high-risk groups to receive the vaccine, especially after the COVID-19 experience. In fact, multiple factors have been associated with the phenomenon of vaccine reluctance including concerns about their potential side effects, mistrust of medical professionals or the healthcare system, a lack of knowledge, and the impact of disinformation, among others [2,10,11]. These factors have also contributed to increased anxiety in the general population, especially in the first months of MPOX re-emergence [2,12]. In this light, the WHO reported that one of the challenges in preventing the re-emergence of MPOX was a lack of knowledge about this disease, particularly among healthcare providers [13].

Thus, the first step in changing public views and behaviors is to address their knowledge. In fact, adequate knowledge could influence positively attitudes, and attitudes based on substantial knowledge are better at predicting behavior than attitudes based on incomplete or inaccurate information [9,14]. Consequently, raising knowledge levels represents an essential measure in fighting against conspiracy theories and understanding health-seeking behaviors such as adherence to preventive measures [9,14]. To this end, multiple studies were carried out to assess the level of knowledge and adherence to a probable future MPOX vaccine targeting multiple categories including the general public, healthcare staff, students in general, and health studies students in particular [9,10,12,13,14,15,16,17,18,19]. These studies shared common findings including a high level of worry, unsatisfactory knowledge and awareness levels, and a medium level of vaccine acceptance. Unfortunately, university students were associated with the lowest levels of knowledge [2]. Knowing their important role in spreading knowledge and awareness among the public, educating them could be a useful strategy in light of the possible emergence of any pandemic [20]. Moreover, the role of students of Health and Life schools as future healthcare providers is of particular interest [9,20].

Thus, the purpose of this work is to assess Algerian Health and Life Science students’ knowledge of MPOX and to determine their attitude toward its probable vaccination, which would provide baseline information about MPOX knowledge and vaccination intention in Algeria.

## 2. Materials and Methods

### 2.1. Study Design

An online-based cross-sectional survey was carried out between 16 December 2022 and 2 March 2023, targeting all Algerian Health and Life Sciences students to gauge their knowledge about MPOX and to describe their attitude toward the MPOX vaccine. Direct contact and social media platforms (including WhatsApp and Facebook) were used to issue invitations to participate in an anonymous online survey by answering a Google Forms self-administered questionnaire (SAQ). In addition, no financial inducements or compensation were given (voluntary participation). With the purpose of minimizing information bias and the Hawthorne effect, the participants’ identities were kept anonymous.

Prior to enrollment, all participants had to provide their e-consent. They were able to leave the questionnaire at any time they wished. The survey was carried out in accordance with the cross-sectional studies STROBE (Strengthening the Reporting of Observational Studies in Epidemiology) standards [21]. The SAQ, which was administered in Arabic and French, was based on earlier studies that looked at knowledge about MPOX and attitude toward its vaccine [19,22,23,24,25,26]. It has 23 multiple-choice items that were divided into four parts. Information on socio-demographic (age, sex, marital status, and living area), educational (specialty, level of education), and vaccination (uptake of flu and COVID-19 vaccines) variables were included in the first part. In the second part, the knowledge level was evaluated using 22 items with “yes/no/I do not know” replies. The attitude toward MPOX vaccines and the willingness to pay were described in the third section using “yes/no” responses. Finally, to assess vaccination beliefs, a 7-item previously validated Vaccine Conspiracy Beliefs Scale (VCBS) using a Likert scale with 7 points was employed in the fourth part [26].

In order to gauge the final level of knowledge, each of the 22 items received a value of one (1) for a correct answer and a value of zero (0) for an incorrect answer (responses with “I do not know” were considered incorrect). Higher scores indicate greater knowledge of MPOX. The 22 items’ scores were totaled up to create an ultimate score that varied from 0 to 23. The mean level (estimated at 9.42) was used to classify the knowledge level as high or low. Respondents with a score of knowledge > 9.42 were deemed to possess a high level of knowledge and vice versa.

The VCBS received a minimum value of 1 for the “strongly disagree” response and a maximum value of 7 for the “strongly agree” response, where higher VCBS represented high acceptance of vaccination conspiracies. The VCBS’s internal consistency was demonstrated through its 0.937 Cronbach’s alpha value [26].

### 2.2. Statistical Analysis

All statistical analyses were conducted using Statistical Package for the Social Sciences (SPSS) version 22.0 (SPSS Inc. Chicago, IL, USA, 2011). The score of knowledge was firstly provided as numbers (n), and percentages (%) or mean ± standard deviation (SD). The relationship between the dependent and the independent variables was assessed using Chi-squared (χ^2^) or Fisher (indicated by an asterisk*) tests. The VCBS was reported as a mean ± SD (estimated at 26.6 ± 11.2).

After that, the hypothesized related factors with MPOX knowledge and attitude regarding MPOX vaccines were evaluated using logistic regression. The statistical analyses were investigated using a threshold of significance (Sig.) of 0.05 and a confidence interval (CI) of ninety-five percent (95%).

## 3. Results

### 3.1. Demographic, Educational, and Vaccine-Status Features

In the current study, 202 respondents completed the questionnaire during the investigation’s time period. After removing six incomplete responses, the remaining (196) responses were used for the analysis.

The demographics, education, and vaccine status of the students are displayed in Table 1. Our results showed that the most represented categories were females (85.7%), singles (95.4%), and those living in urban areas (89.3%), while more than half of the respondents (55.1%) were under 20 years old.

Regarding their education, students of Medicine (64.3%) were the most represented followed distantly by Pharmacy (11.7%), and Natural and Life Sciences (11.7%) students. They were mostly in the first year of their university curricula (55.1%).

When asked about their vaccination status, only 21.9% stated that they had received the COVID-19 vaccine, while less than one-third (30.1%) received a seasonal flu vaccine (Table 1).

### 3.2. MPOX Knowledge

The results of this study showed that 77.6% of the participants had heard about the MPOX pandemic of 2022, while 68.4% reported having a prior knowledge about this disease before its re-emergence in 2022.

Moreover, 54.6% of the respondents thought that this disease is not adequately covered by the media, while 36.7% thought that it is adequately covered. Similarly, 41.8% of respondents stated they were concerned about the spread of this disease, while 39.3% said they were indifferent, and 18.9% said they were not concerned.

Regarding level of knowledge, our findings showed that the average percentage of right answers was 42.8%. This rate reflects a score of knowledge of 9.42 ± 4.79 (from 22 possible points). Additionally, 7 of the 22 items had more than 50% of correct responses. These items are mainly related to the absence of MPOX cases in Algeria (63.3%), its high frequency in Western and Central African countries (53.1%), its viral etiology (66.3%), and its main signs including rash (77.6%), papules (59.2%), and pustules (60.2%) (Table 2).

**Figure 1:**
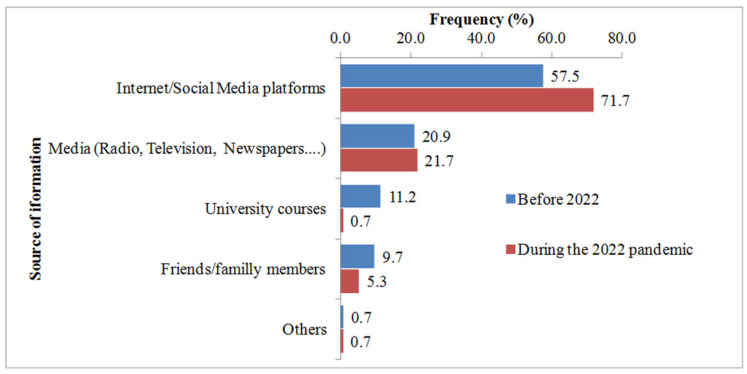
Sources of information on MPOX of the surveyed population.

When asked about their sources of information, the respondents cited the internet and social media platforms, in addition to media, as the main sources used to acquire information about MPOX (Figure 1).

#### Knowledge-Related Factors

The findings indicated that students over 30 years old (84.6%) showed higher levels of knowledge (Sig. = 0.007). Similar results were observed among students of veterinary sciences (81.3%, Sig. = 0.004) while those of medicine and dental sciences (39.7%, Sig. = 0.013) were characterized by the lowest level of knowledge. In addition, students in the first to the third years of study showed lower levels of knowledge (41.2%) than their counterparts but without a statistical difference (Sig. = 0.061).

Regarding vaccination, our results showed that individuals with previous uptake of a flu vaccine were inversely linked to high levels of knowledge (35%, Sig. = 0.042), while no statistical difference was observed for COVID-19 vaccine uptake (Sig. = 0.260).

Results of the logistic regression showed that students of 20–30 years showed a lower high knowledge OR score (OR: 0.11; CI95%: 0.02–0.79) than those over the age of 30 years. Additionally, while students of veterinary sciences showed the highest OR of high knowledge (OR: 6.71; CI95%: 1.23–36.77), being vaccinated against seasonal flu decreased the odds of high knowledge (OR: 0.42; CI95%: 0.21–0.85) (Table 3).

### 3.3. Vaccination Intention

Asked about their intention regarding a probable recommended MPOX vaccine in the near future, 48.5% of the surveyed students declared their willingness to be vaccinated. Moreover, 70.5% would be willing to cover the costs of the vaccination if it is not freely available.

Results of the univariate analysis showed that students of Medicine and Dental Science, those of the fourth and the fifth year of study, showed higher rates of willingness than their counterparts. Inversely, students of Natural and Life Sciences and those having a high VCBS showed the lowest levels. The latter factors were the only factors confirmed by logistic regression to be connected to vaccine reluctance (Table 4).

## 4. Discussion

The present survey aimed to analyze the level of awareness and knowledge of Algerian Health and Life Science students about MPOX and their attitude toward its immunization. As far as we are aware, this is the first study to document MPOX-related knowledge among students in Algeria.

The main conclusions of this study can be summarized as follows. First, more than one-fifth (22.4%) of the questioned students were unaware of the ongoing 2022 MPOX multi-country outbreak. Second, a low level of knowledge with multiple gaps about the epidemiology, the etiology, and the clinical manifestations of MOPX was observed. Third, nearly half (48.5%) of the surveyed students were in favor of vaccination if recommended in the country and 70.5% among them showed their willingness to pay for the vaccine if it was not provided for free.

The first surprising result of this study is the fact that 22.4% of the respondents had not heard of the MPOX pandemic even though the survey was conducted nearly six months after its emergence. This finding could be explained by the absence of any reported cases of MPOX in Algeria. In a previous study conducted earlier among healthcare staff in Algeria, 91.9% were aware of this pandemic [19]. In line with this study, 54.6% of the respondents thought that this disease lacks media coverage and 41.8% expressed their concern about the propagation of the disease. The same observation was reported in different countries (with different levels) [15,17,20,27,28] where people were afraid to be infected by the disease [16] or that the disease would progress to becoming a pandemic [12]. These observations are mainly related to the drastic social, health, and economic consequences engendered by the COVID-19 pandemic [10,12].

Regarding knowledge about this disease, even though multiple scales have been used and multiple categories have been targeted (students, medical students, healthcare workers, the general population) in different countries, the common finding is the unsatisfactory level of knowledge about MPOX with multiple gaps [9,10,17,18,29,30,31,32,33,34,35]. Certain knowledge gaps were also reported among medical students in endemic countries [36].

For the knowledge-linked factors, even age and educational attainment have been reported as determinants in most studies [14], other factors were also reported (i.e., professional standing, information source, and belief in conspiracy theories).

In our study, the effect of age was apparent, in which students aged more than 30 years showed the highest level of knowledge (OR: 0.11; CI95%: 0.02–0.79 for those aged between 20 and 30 years old). In the same way, Sallam et al. [9] and Alshahrani et al. [31] showed that students aged more than 21 years old showed higher levels of knowledge than their younger counterparts. Meanwhile, Lin et al. [20] found that dental clinical students were more knowledgeable about MPOX than preclinical ones.

Surprisingly, veterinary students have shown higher levels of knowledge (OR: 6.71; CI95%: 1.23–36.77) compared to those of the other specialties. These findings could be explained by the fact that most of the students of medicine surveyed were in their first few years of study, whereas most of the veterinary students surveyed were in their fourth or fifth year of study. As a comparison, Kumar et al. [37] in Pakistan found that students of pharmaceutical and medical sciences showed higher levels of knowledge than students of biological sciences or those of other domains.

Another factor, which was incomprehensibly associated with the level of knowledge, was the seasonal flu vaccination status. In fact, students who were vaccinated against seasonal flu showed the lowest levels of knowledge about MPOX (OR: 0.42; CI95%: 0.21–0.85).

Conversely, no link was observed between vaccination status against seasonal flu or COVID-19 and the attitude toward MPOX vaccination among the surveyed students. This is contrary to the results of Winters et al. [38], which showed that COVID-19 vaccine uptake was a strong predictor of MPOX vaccination willingness in the US general population. Similarly, Riccò et al. [17] showed that individuals vaccinated against seasonal influenza are more in favor of the MPOX vaccine. In a study conducted earlier in Algeria, COVID-19 vaccination was a determinant factor for the acceptance of MPOX vaccination [19].

Interestingly, students having a high mean of VCBS showed the lowest level of vaccine acceptance. The VCBS, which was first adopted for HPV vaccine reluctance [26], was reported to be a good scale for vaccination hesitancy, which was subsequently confirmed for the Influenza [39] and COVID-19 vaccines [40].

Finally, it is important to emphasize the medium and the encouraging level (48.5%) of willingness to be vaccinated among the surveyed students, which is higher than the level of 38.7% reported previously among healthcare workers in Algeria [19]. This rate is not far from the global level of acceptance and very close to the level of acceptance in Asian countries [2,11].

Undoubtedly, this study was limited by multiple factors: First, the convenience sampling method carried the risk of selection bias. In addition, the online nature of the survey, especially conducted using social media platforms, may affect certain responses and lead to a bias in answers. (i.e., the media through which information is received). Second, the sample, which was composed of a majority of medical students, increases the selection bias and should be taken into account when attempting to generalize the study’s findings. Third, the sample size could be considered as another limitation, especially in sub-group analysis. Fourth, the self-reported nature of the responses should be considered, acknowledging the potential for social desirability bias. Finally, the cross-sectional nature of this study makes its finding a snapshot of the current situation about knowledge and vaccine intention, which may change over time.

## 5. Conclusions

The findings of the present study reveal a poor level of knowledge of Algerian Health and Life Sciences students about MPOX with multiple knowledge gaps regarding its epidemiology, etiology, and clinical signs. They also show that despite this poor level of knowledge, a satisfactory medium level of acceptance of MPOX vaccination exists, not far from the global rate of acceptance. This is especially noteworthy given that we know that, to date, no cases of MPOX have been reported in Algeria. Thus, intensifying educational and awareness programs is required to increase the level of knowledge about this disease and show the importance of MPOX vaccination, an approach that could be useful in prevention strategies and fighting against the phenomenon of vaccine hesitancy often mediated by belief in conspiracy theories.

## Figures and Tables

**Table 1:** Demographics, education, and vaccine status of the respondents.

Variable		Number	Frequency (%)
Age	Under 20 years	108	55.1
	20–29 years	75	38.3
	Over 30 years	13	6.6
Sex	Females	168	85.7
	Males	28	14.3
Marital status	Married	9	4.6
	Single	187	95.4
Living area	Rural	21	10.7
	Urban	175	89.3
Specialty	Medicine	126	64.3
	Natural and Life Sciences	23	11.7
	Pharmacy	23	11.7
	Veterinary Sciences	16	8.2
	Dental Sciences	5	2.6
	Paramedical Sciences	3	1.5
Educational level	First year	108	55.1
	Second year	10	5.1
	Third year	13	6.6
	Fourth year	8	4.1
	Fifth year	27	13.8
	Sixth year	16	8.2
	Seventh year	14	7.1
Influenza vaccine	Yes	60	30.6
	No	136	69.4
COVID-19 vaccine	Yes	43	21.9
	No	153	78.1

**Table 2:** Average correct responses for the different items.

Item	Correct Responses (%)
1. MPOX is prevalent in North African countries	48.5
2. MPOX is prevalent in Western and Central Africa	53.1
3. There are many MPOX cases in Algeria	63.3
4. MPOX is a viral disease infection	66.3
5. MPOX is a bacterial disease infection	59.7
6. MPOX is easily transmitted from human to human	19.9
7. MPOX can be transmitted through a bite of an infected monkey	45.9
8. Sexual transmission is the main route of transmission of MPOX	29.1
9. Monkeys are the main reservoir of MPOX virus	15.8
10. MPOX and smallpox have similar signs and symptoms	48.5
11. Flu-like syndrome is one of the early signs or symptoms of MPOX	34.7
12. Rashes on the skin are one of the signs or symptoms of MPOX	77.6
13. Papules on the skin are one of the signs or symptoms of MPOX	59.2
14. Vesicles on the skin are one of the signs or symptoms of MPOX	44.9
15. Pustules on the skin are one of the signs or symptoms of MPOX	60.2
16. Lymphadenopathy (swollen lymph nodes) is one clinical sign or symptom that could be used to differentiate MPOX and smallpox cases	37.8
17. Diarrhea is one of the signs or symptoms of MPOX	19.4
18. Antivirals are required in the management of MPOX patients	23.5
19. Antibiotics are required in the management of MPOX patients	25.0
20. The case fatality rate of MPOX is high	43.9
21. Vaccination is available for MPOX in Algeria	38.8
22. There is a smallpox vaccine that can be used for MPOX	27.6
Average	42.8

**Table 3:** Knowledge-related factors of the surveyed students.

		High (%)	Low (%)	*Sig*.	OR (CI95%)	*Sig*.
Age	Under 20 years	45 (41.7)	63 (58.3)	0.186	0.36 (0.05–2.81)	0.332
	20–29 years	34 (45.3)	41 (54.7)	0.897	0.11 (0.02–0.79)	**0.028**
	Over 30 years	11 (84.6)	2 (15.4)	0.007 *	.	.
Sex	Females	76 (45.2)	92 (54.8)	0.640	1.45 (0.54–3.91)	0.459
	Males	14 (50)	14 (50)		.	.
Living area	Rural	12 (57.1)	9 (42.9)	0.357 *	1.53 (0.57–4.10)	0.400
	Urban	78 (44.6)	97 (55.4)		.	.
Specialty	Pharmacy	10 (43.5)	13 (56.5)	0.803	1.11 (0.38–3.25)	0.855
	Veterinary Sciences	13 (81.3)	3 (18.8)	0.004 *	6.71 (1.23–36.77)	**0.028**
	NLS	12 (52.2)	11 (47.8)	0.522	3.45 (0.93–12.84)	0.065
	Medicines (dental)	52 (39.7)	79 (60.3)	**0.013**	.	.
Educational level	4–5 years	21 (60)	14 (40)	0.065	1.52 (0.42–5.56)	0.524
	6–7 years	15 (50)	15 (50)	0.626	2.04 (0.54–7.66)	0.293
	1–3 years	54 (41.2)	77 (58.8)	0.061	.	.
Influenza vaccine	Yes	21 (35)	39 (65)	**0.042**	0.42 (0.21–0.85)	**0.016**
	No	69 (50.7)	67 (49.3)		.	.
COVID-19 vaccine	Yes	23 (53.5)	20 (46.5)	0.260	1.78 (0.73–4.30)	0.203
	No	67 (43.8)	86 (56.2)		.	.

NLS, Natural and Life Science. Bold character indicates significant results with a statistical difference (Sig. < 0.05) using Chi-squared or Fisher (indicated by an Asterisk (*)) tests.

**Table 4:** Willingness-to-vaccinate determinants in the surveyed population.

Variable		Yes (%)	No (%)	*Sig*.	OR (CI95%)	*Sig*.
Age	Under 20 years	58 (53.7)	50 (46.3)	0.104	2.55 (0.42–15.36)	0.306
	20–29 years	33 (44)	42 (56)	0.324	3.03 (0.66–13.84)	0.152
	Over 30 years	4 (30.8)	9 (69.2)	0.253 *	.	.
Sex	Female	81 (48.2)	87 (51.8)	0.861	0.83 (0.37–2.14)	0.706
	Males	14 (50)	14 (50)			
Living area	Rural	8 (38.1)	13 (61.9)	0.314	0.77 (0.28–2.10)	0.605
	Urban	87 (49.7)	88 (50.3)		.	.
Specialty	Veterinary Sciences	5 (31.3)	11 (68.8)	0.196 *	0.47 (0.10–2.17)	0.333
	NLS	5 (21.7)	18 (78.3)	**0.006**	0.14 (0.03–0.59)	**0.008**
	Paramedical Sciences	1 (33.3)	2 (66.7)	1 *	0.56 (0.03–9.38)	0.688
	Pharmacy	9 (39.1)	14 (60.9)	0.340	0.481 (0.16–1.45)	0.193
	Medicine (dental)	75 (57.3)	56 (42.7)	**0.0005**	.	.
Educational level	4–5 years	12 (34.3)	23 (65.7)	**0.064**	1.273 (0.34–4.83)	0.722
	6–7 years	15 (50)	15 (50)	0.855	1.14 (0.31–4.17)	0.842
	1–3 years	68 (51.9)	63 (48.1)	0.171	.	.
Influenza vaccine	Yes	34 (56.7)	26 (43.3)	0.127	1.90 (0.95–3.78)	0.069
	No	61 (44.9)	75 (55.1)		.	
COVID-19 vaccine	Yes	23 (53.5)	20 (46.5)	0.456	1.49 (0.63–3.56)	0.367
	No	72 (47.1)	81 (52.9)		.	.
Knowledge score	High	39 (43.3)	51 (56.7)	0.185	0.85 (0.45–1.61)	0.618
	Low	56 (52.8)	50 (47.2)		.	.
VCBS scale	High	41 (39.8)	62 (60.2)	**0.011**	0.476 (0.26–0.89)	**0.019**
	Low	54 (58.1)	39 (41.9)		.	.

NLS, Natural and Life Science. Bold character indicates significant results with a statistical difference (Sig. < 0.05) using Chi-squared or Fisher (indicated by an Asterisk (*)) tests.

## Data Availability

Data can be made available by the authors upon request.
